# STAT3/miR-135b/NF-κB axis confers aggressiveness and unfavorable prognosis in non-small-cell lung cancer

**DOI:** 10.1038/s41419-021-03773-x

**Published:** 2021-05-14

**Authors:** Jinlin Zhao, Xin Wang, Zeyun Mi, Xiangli Jiang, Lin Sun, Boyu Zheng, Jing Wang, Maobin Meng, Lu Zhang, Zhongqiu Wang, Junwei Song, Zhiyong Yuan, Zhiqiang Wu

**Affiliations:** 1 Department of Radiation Oncology, Tianjin Medical University Cancer Institute & Hospital, Key Laboratory of Cancer Prevention and Therapy, National Clinical Research Center for Cancer, Tianjin’s Clinical Research Center for Cancer, 300060 Tianjin, China; 2 Department of Biochemistry and Molecular Biology, College of Basic Medical Science, Tianjin Medical University, 300070 Tianjin, China; 3 Department of Thoracic Medical Oncology, Tianjin Medical University Cancer Institute & Hospital, 300060 Tianjin, China; 4 Department of Pathology, Tianjin Medical University Cancer Institute & Hospital, 300060 Tianjin, China; 5 Guangdong Key Laboratory for Genome Stability and Human Disease Prevention, Department of Biochemistry and Molecular Biology, Department of Pathogen Biology, Guangdong Provincial Key Laboratory of Regional Immunity and Diseases, Shenzhen University School of Medicine, 518060 Shenzhen, Guangdong China

**Keywords:** miRNAs, Non-small-cell lung cancer

## Abstract

Non-small-cell lung cancer (NSCLC) is one of the most commonly diagnosed cancers worldwide but has limited effective therapies. Uncovering the underlying pathological and molecular changes, as well as mechanisms, will improve the treatment. Dysregulated microRNAs (miRNAs) have been proven to play important roles in the initiation and progression of various cancers, including NSCLC. In this manuscript, we identified microRNA-135b (miR-135b) as a tumor-promoting miRNA in NSCLC. We found that miR-135b was significantly upregulated and that its upregulation was associated with poor prognosis in NSCLC patients. miR-135b was an independent prognostic factor in NSCLC. Overexpressing miR-135b significantly promoted the aggressiveness of NSCLC, as evidenced by enhanced cell proliferation, migration, invasion, anti-apoptosis, and angiogenesis in vitro and in vivo, and knockdown of miR-135b had the opposite effects. Mechanistically, our results reveal that miR-135b directly targets the 3′-untranslated region (UTR) of the deubiquitinase CYLD, thereby modulating ubiquitination and activation of NF-κB signaling. Moreover, we found that interleukin-6 (IL-6)/STAT3 could elevate miR-135b levels and that STAT3 directly bound the promoter of miR-135b; thus, these findings highlight a new positive feedback loop of the IL-6/STAT3/miR-135b/NF-κB signaling in NSCLC and suggest that miR-135b could be a potential therapeutic target for NSCLC.

## Introduction

Most, if not all, solid tumors are infiltrated with immune and inflammatory cells^1^. In 1863, Rudolf Virchow initially proposed a functional relationship between inflammation and cancer based on his observation that a high number of leukocytes were present in tumor samples^2^. An increasing number of studies have proven that chronic inflammation promotes tumor progression. Inflammation is a hallmark of cancer and is pivotal during cell transformation, invasion, metastasis, and treatment resistance^3–5^. It is estimated that ~15% of human cancers are associated with chronic infections and inflammation^4^. Therefore, uncovering the signaling pathways and underlying mechanisms involved in cancer-related inflammation will help to find novel targets for cancer prevention and treatment.

Several cellular pathways have been proven to take part in cancer-related inflammation, among which the most prominent is the NF-κB pathway and interleukin 6 (IL-6)/STAT3 signaling. NF-κB is constitutively activated and promotes aggressiveness in a wide variety of cancers^6,7^. Upon signal induction, for example, TNF-α or IL-1β treatment, a series of signaling intermediaries become ubiquitinated, for example, TRAFs, RIP1, and NEMO are modified with K63-linked polyubiquitination (poly-Ub), which facilitates activation of the IKK complex and is essential for subsequent nuclear translocation and full activation of NF-κB^8–10^. Deubiquitinase that removes K63-linked poly-Ub from signaling intermediaries, such as CYLD and A20, had been proven to negatively regulate NF-κB signaling activation and tumor progression^11,12^. Additionally, STAT3 signaling is hyperactivated in the majority of human cancers and a well-established intrinsic pathway driving inflammation, cellular transformation, survival, proliferation, invasion, angiogenesis, metastasis, and immune evasion in cancer^13,14^. IL-6 is a major cytokine activating STAT3 and is proposed to be related to advanced-stage disease and decreased survival in cancer^15^. Moreover, NF-κB and STAT3 also collaborate and engage in crosstalk in cancer. Targeting these pathways has resulted in favorable oultcomes in cancer treatment^16,17^.

MicroRNAs (miRNAs) are a class of small non-coding RNAs consisting of 17–25 nucleotides. miRNAs target the 3′-untranslated region (UTR) of mRNAs or other non-coding RNAs and interact with Argonaute (AGO) proteins to form the RNA-induced silencing complex (RISC), inhibiting the expression of target genes or mediating their degradation^18^. Dysregulated miRNAs participate in various cellular pathways and regulate proliferation, apoptosis, invasion, and metastasis in various tumors^19^. MicroRNA-135b (miR-135b) has been reported to be aberrantly expressed and to play a role in the progression of a variety of cancers, such as gastric cancer^20^, breast cancer^21–23^, lymphoma^24^, and lung cancer^25,26^, by targeting different genes and pathways. However, in non-small-cell lung cancer (NSCLC), the role of miR-135b in tumor progression remains controversial. Whether miR-135b affects cancer-related inflammatory pathways is currently unknown.

In this research, we demonstrated that miR-135b was significantly upregulated and that its expression was negatively correlated with the prognosis of NSCLC patients. In addition, miR-135b was found to promote NSCLC cell proliferation, migration, invasion, anti-apoptosis, and angiogenesis in vitro and in vivo. Furthermore, we revealed that miR-135b targets and downregulates CYLD and thereby activates the NF-κB pathway. Finally, we determined that IL-6/STAT3 signaling transactivates miR-135b and that STAT3 directly binds the promoter of miR-135b. Collectively, our results prove the tumor-promoting role and underlying mechanism(s) of miR-135b in NSCLC.

## Results

### miR-135b is significantly upregulated and correlated with poor prognosis of patients in NSCLC

**Fig. 1:**
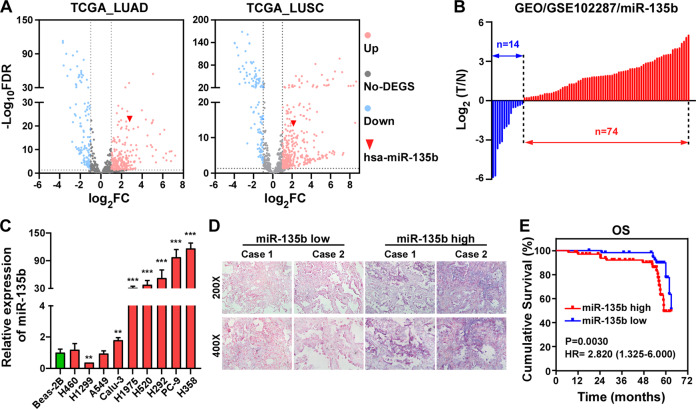
miR-135b is elevated and associated with poor prognosis of patients in NSCLC. **A** Volcano plots analyzing differential expressions of miRNAs in LUAD and LUSC datasets from the TCGA database. **B** Analysis of the expression of miR-135b in NSCLC dataset from the NCBI/GEO database. **C** Real-time PCR analysis of miR-135b level in lung epithelial cell line, Beas-2B, and NSCLC cell lines. Error bars represent the mean ± SD obtained from three independent experiments. *p* values are calculated by a two-tailed, unpaired *t*-test. **p < 0.01; ***p < 0.001. **D** Representative photographs of in situ hybridization staining of miR-135b in NSCLC tissues. Original magnification: Upper, ×200; Lower, ×400. **E** Kaplan–Meier analysis of overall survival (OS) in 128 NSCLC patients.

First, we mined The Cancer Genome Atlas (TCGA) database (https://cancergenome.nih.gov/) and the NCBI/GEO database (https://www.ncbi.nlm.nih.gov/geo/) to evaluate the expression of miR-135b in NSCLC. As shown in Fig. 1A, miR-135b was one of the most significantly upregulated miRNAs in tumor tissues from both lung adenocarcinoma (LUAD) and lung squamous cell carcinoma (LUSC) patients, especially in LUAD tissues. Compared to paired adjacent nontumor tissue, the majority of tumor tissues showed increased expression of miR-135b (Fig. 1B). Consistently, analyzing the expression of miR-135b in NSCLC cell lines revealed that miR-135b was heterogeneously increased in 6/9 and dramatically upregulated over 30 folds in 5/9 of the analyzed cell lines compared with the normal BEAS-2B cell line (Fig. 1C). Moreover, we detected the expression of miR-135b in a cohort of NSCLC specimens by an in situ hybridization assay. Representative images of NSCLC specimens stained with low or high miR-135b by in situ hybridization are shown in Fig. 1D. The expression of miR-135b did not significantly correlate with the main clinicopathological features, TNM classification, or clinical stage of NSCLC patients (Table S3). Univariate Cox regression analysis showed that the expression of miR-135b was significantly correlated with patient survival status (regression coefficient = 0.830, hazard ratio (HR) = 3.409, 95% CI = 1.501–7.742, *p* = 0.003, Table 1). Multivariate Cox analysis revealed that miR-135b was an independent prognostic factor for NSCLC with an HR of 4.822 (95% CI = 1.978–11.757, *p* = 0.001, Table 1). Furthermore, Kaplan–Meier survival analysis proved that NSCLC patients with high miR-135b expression had shorter overall survival (OS) time than those with low miR-135b expression. The odds ratio (OR) was 2.820 (95% CI, 1.325 to 6.000, *p* = 0.0030, Fig. 1E). The above data suggest that miR-135b is a poor prognostic factor for NSCLC patients.

**Table 1:** Univariate and multivariate analysis of different prognostic parameters in patients with NSCLC by Cox-regression analysis.

Variable	Univariate analysis				Multivariate analysis		
	Regression coefficient (SE)	HR	95% CI	*P* Value	HR	95% CI	*P* Value
Gender	−0.335 (0.381)	0.715	0.339–1.510	0.380			
Age (years)	−0.692 (0.391)	0.501	0.232–1.078	0.077			
Smoke	0.200 (0.373)	1.221	0.588–2.536	0.592			
T classification	0.563 (0.216)	1.756	1.149–2.683	0.009	─	─	0.174
N classification (N0 vs. N1 + N2)	1.188 (0.403)	3.282	1.489–7.233	0.003	4.302	1.914–9.667	<0.001
Clinical stage (I + II vs. III + IV)	1.201 (0.387)	3.324	1.556–7.103	0.002	─	─	0.821
miR-135b (low vs. high)	1.226 (0.419)	3.409	1.501–7.742	0.003	4.822	1.978–11.757	0.001

### MiR-135b enhances the growth and proliferation of NSCLC cells

**Fig. 2:**
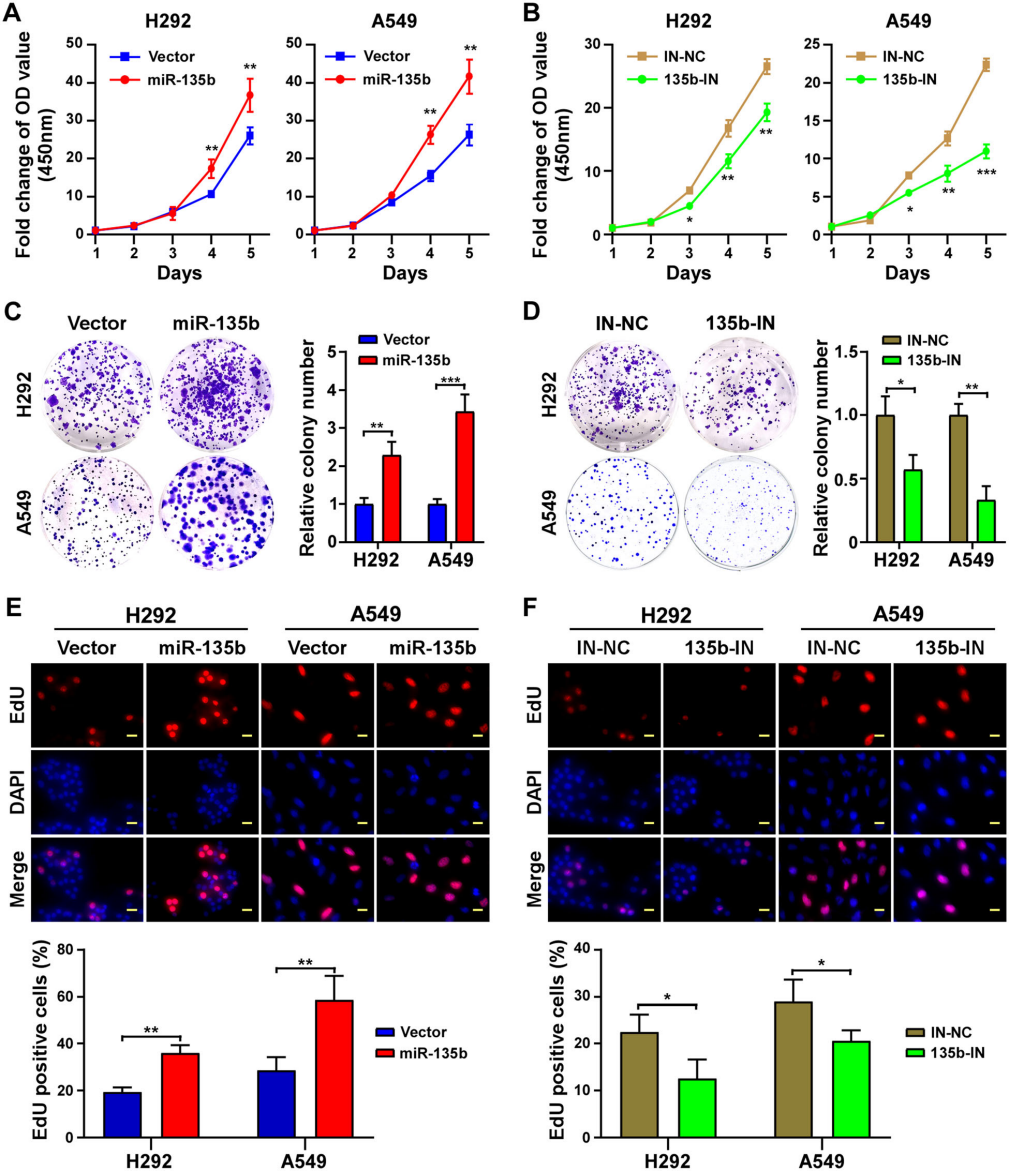
miR-135b enhances growth and proliferation of NSCLC cells in vitro. **A**, **B** CCK-8 assay analysis of cell growth in the indicated stable cell lines. **C**, **D** Representative pictures (Left panel) and quantification (Right panel) of cell colony formation. **E**, **F** Representative pictures (Upper panel) and quantification (lower panel) of EdU staining. EdU was labeled with TRITC (red) and cells were counterstained with DAPI (blue). IN-NC: Inhibiting negative control; 135b-IN: miR-135b inhibiting. Scale bars, 20 μm. Error bars represent the mean±SD obtained from three independent experiments. *p* values are calculated by a two-tailed, unpaired *t*-test. *p < 0.05; **p < 0.01; ***p < 0.001.

The effects of miR-135b on the growth and proliferation of NSCLC cells were evaluated. According to the results of Fig. 1C, the two cancer cell lines, H292 and A549, were included to stably overexpress miR-135b or to have miR-135b knockdown (Fig. S1) and a series of assays were performed. The CCK-8 assay proved that overexpression of miR-135b enhanced while inhibition of miR-135b impaired the growth of both cell lines (Fig. 2A, B). Colony formation assays showed that miR-135b overexpressing cells formed more and larger cell colonies than vector control and inhibiting miR-135b dramatically decreased the cell colony number (Fig. 2C, D). In addition, the EdU incorporation assay showed that miR-135b overexpressing cells incorporated much more EdU, while those with miR-135b knockdown incorporated less EdU (Fig. 2E, F), which further demonstrated the proliferation-promoting role of miR-135b in NSCLC.

### miR-135b promotes cell migration, invasion, anti-apoptosis, and angiogenesis in NSCLC

**Fig. 3:**
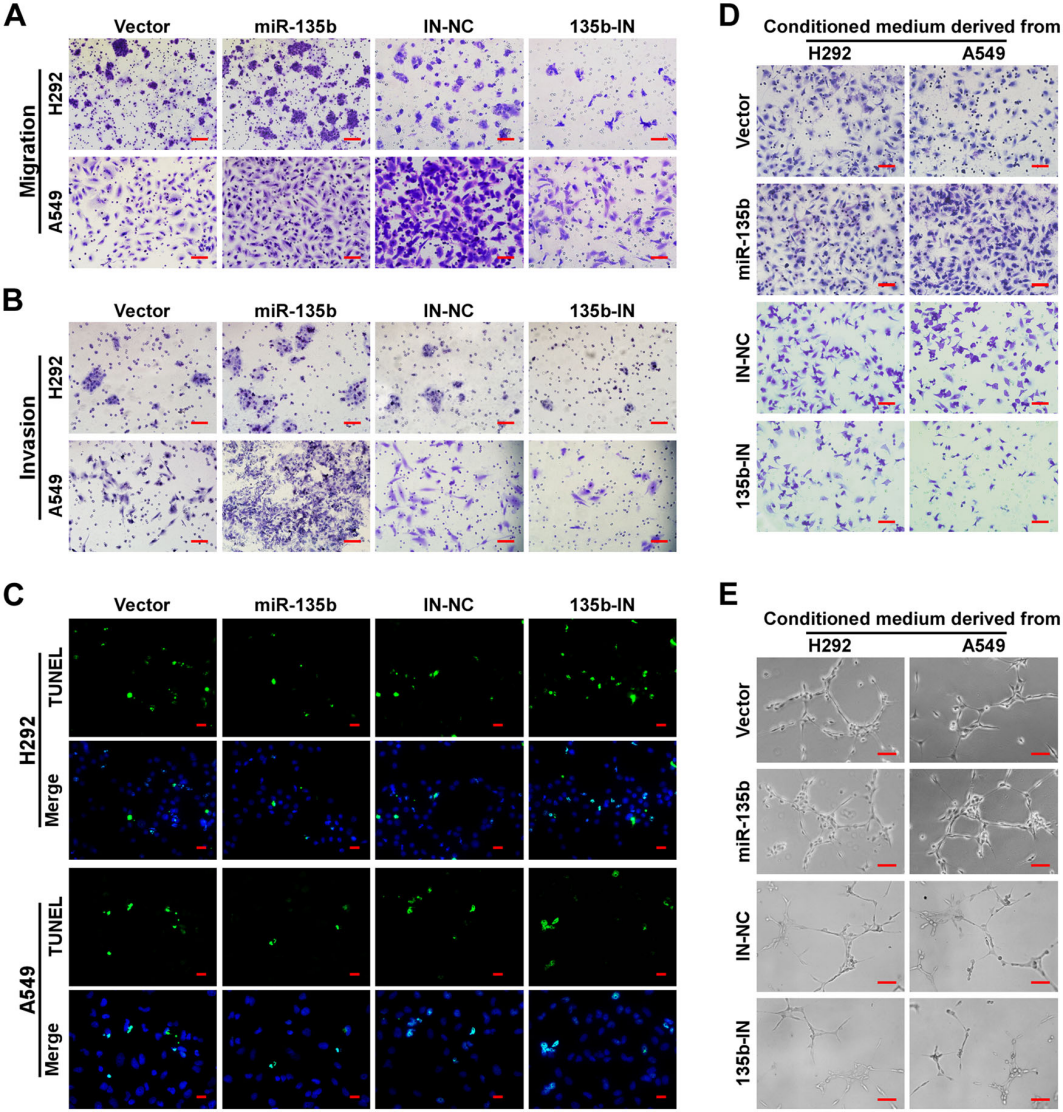
miR-135b promotes cell migration, invasion, anti-apoptosis, and angiogenesis in NSCLC. **A**, **B** Representative pictures of cell migration (**A**) and invasion (**B**) in H292 and A549cells analyzed by transwell assay. Scale bars, 100 μm. **C** Representative pictures of cell apoptosis after treatment with cisplatin for 24 h analyzed by TUNEL staining (green). Cells were counterstained with DAPI. Scale bars, 20 μm. **D** Cell migration was assessed by culturing HUVECs with conditioned medium collected from the indicated NSCLC cells. Scale bars, 100 μm. **E** Representative images of tubule formation by HUVECs cultured in Matrigel-coated plates with conditioned medium collected from the indicated NSCLC cells. Scale bars, 50 μm.

Next, we detected the effects of miR-135b on cell migration, invasion, apoptosis, and angiogenesis. Data from transwell assays with chambers coated with or without Matrigel showed that there were more cells present on the bottom of the chambers for miR-135b overexpressing cells and fewer cells for the miR-135b-IN group cells than for the control cells (Figs. 3A, B, and S2A, B). These results indicate that miR-135b significantly increases the migration and invasion of NSCLC cells. Additionally, overexpression of miR-135b significantly inhibited cell apoptosis as indicated by a smaller portion of cells with TUNEL positivity (Figs. 3C and S2C), fewer dead cells, and decreased cell debris observed under a bright-field microscope (Fig. S2D), as well as decreased expression of cleaved PARP (c-PARP, Fig. S2E). However, inhibiting miR-135b produced the opposite results. Moreover, overexpression of miR-135b strongly enhanced the ability of NSCLC cells to induce migration and tubule formation by HUVECs (Figs. 3D, E and S2F), and miR-135b knockdown produced the opposite results.

### miR-135b activates NF-κB signaling

**Fig. 4:**
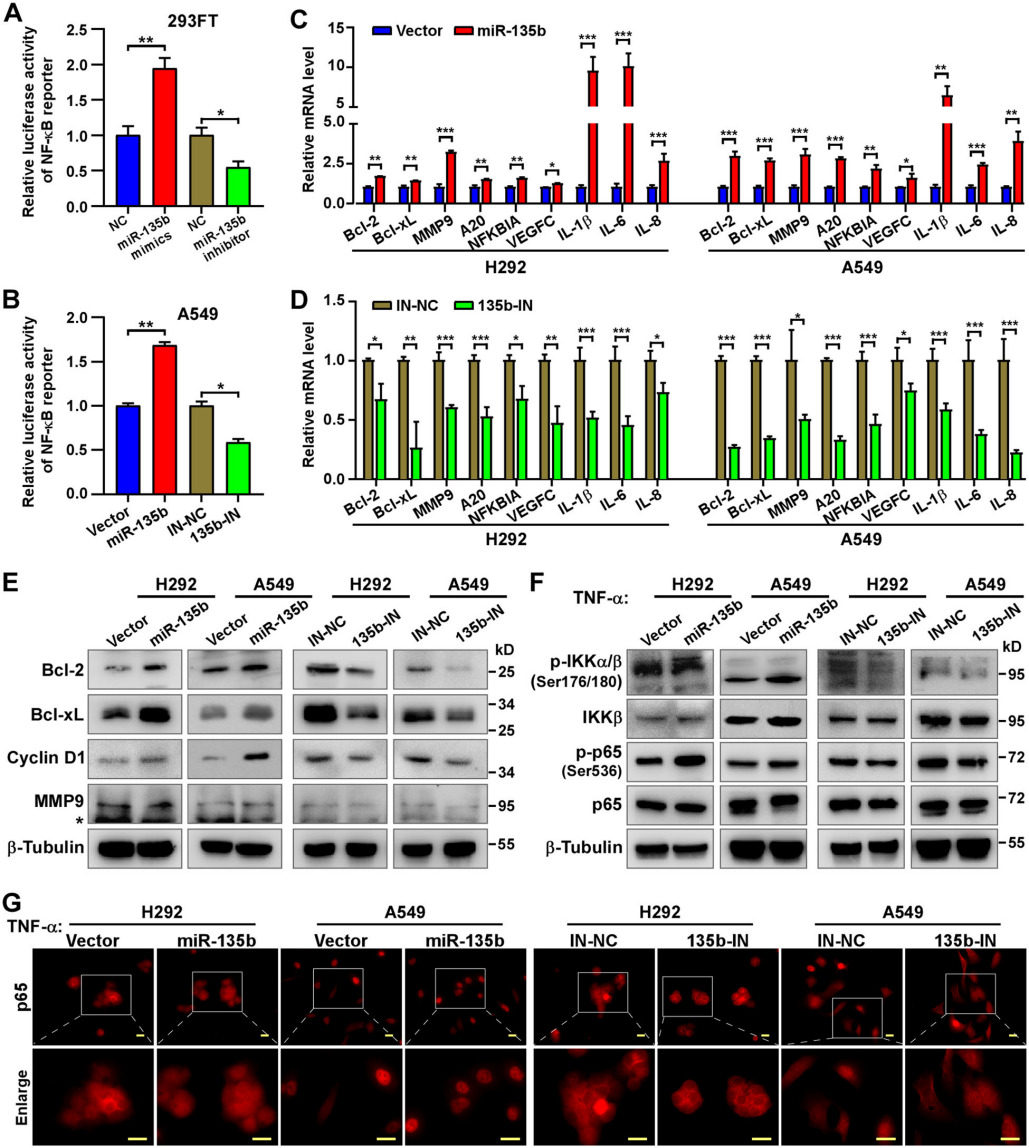
miR-135b activates the NF-κB signaling. **A**, **B** Activity of the NF-κB luciferase reporter gene in 293FT (**A**) transfected with miR-135b mimics, miR-135b inhibitors and corresponding negative control (NC) and in A549 stable cell lines (**B**). **C**, **D** Real-time PCR analysis of the mRNA expression of NF-κB downstream genes in the indicated cells. **E** Western blotting detecting the protein level of NF-κB downstream genes, Bcl-2, Bcl-xL, Cyclin D1, and MMP9 in indicated cells. β-tubulin served as a loading control. *Non-specific band. **F** Western blotting detecting the protein level of NF-κB signaling, p65, phospho-p65 (p-p65), IKKβ, phospho-IKKα/β (p-IKKα/β), in the indicated cells stimulated with TNF-α (10 ng/ml) for 20 min. β-tubulin served as a loading control. **G** Representative images of immunofluorescence staining of p65 (labeled with TRITC) in the indicated cells stimulated with TNF-α (10 ng/ml) for 20 min. Scale bars, 20 μm. Error bars represent the mean ± SD obtained from three independent experiments. *p* values are calculated by a two-tailed, unpaired *t*-test. *p < 0.05; **p < 0.01; ***p < 0.001.

The NF-κB signaling pathway is well known to play critical roles in cell proliferation, migration, invasion, anti-apoptosis, angiogenesis, and so on during tumor progression. Previously we have proved the involvement of NF-κB signaling in different types of tumors^27,28^. Herein, we performed a luciferase reporter assay to investigate whether miR-135b could activate NF-κB signaling. Indeed, both 293FT cells transiently transfected with miR-135b mimics and A549 cells induced to have stable ectopic overexpression of miR-135b showed higher NF-κB reporter luciferase activity than controls and inhibiting miR-135b decreased NF-κB reporter activity (Fig. 4A, B). Additionally, overexpression of miR-135b significantly increased the mRNA levels of NF-κB downstream genes, such as Bcl-2, Bcl-xL, MMP9, A20, NFKBIA, VEGFC, IL-1β, IL-6, and IL-8, analyzed by qRT-PCR (Fig. 4C, D), as well as protein levels of Bcl-2, Bcl-xL, cyclin D1, and MMP9 analyzed by western blotting. Furthermore, inhibiting miR-135b had the opposite effects (Fig. 4E). Next, the effects of miR-135b on NF-κB pathway signal transduction were investigated. As shown in Fig. 4F, overexpression of miR-135b increased while inhibition of miR-135b decreased phosphorylation of IKK and NF-κB/p65 upon TNF-α treatment in both H292 and A549 cells. Moreover, immunofluorescence staining revealed that overexpression of miR-135b promoted nuclear translocation of NF-κB/p65, which is essential for transactivating downstream genes, and vice versa (Figs. 4G and S3). Overall, our results suggest that miR-135b serves as an activator of the NF-κB pathway.

### miR-135b activates NF-κB signaling by targeting the deubiquitinase CYLD

**Fig. 5:**
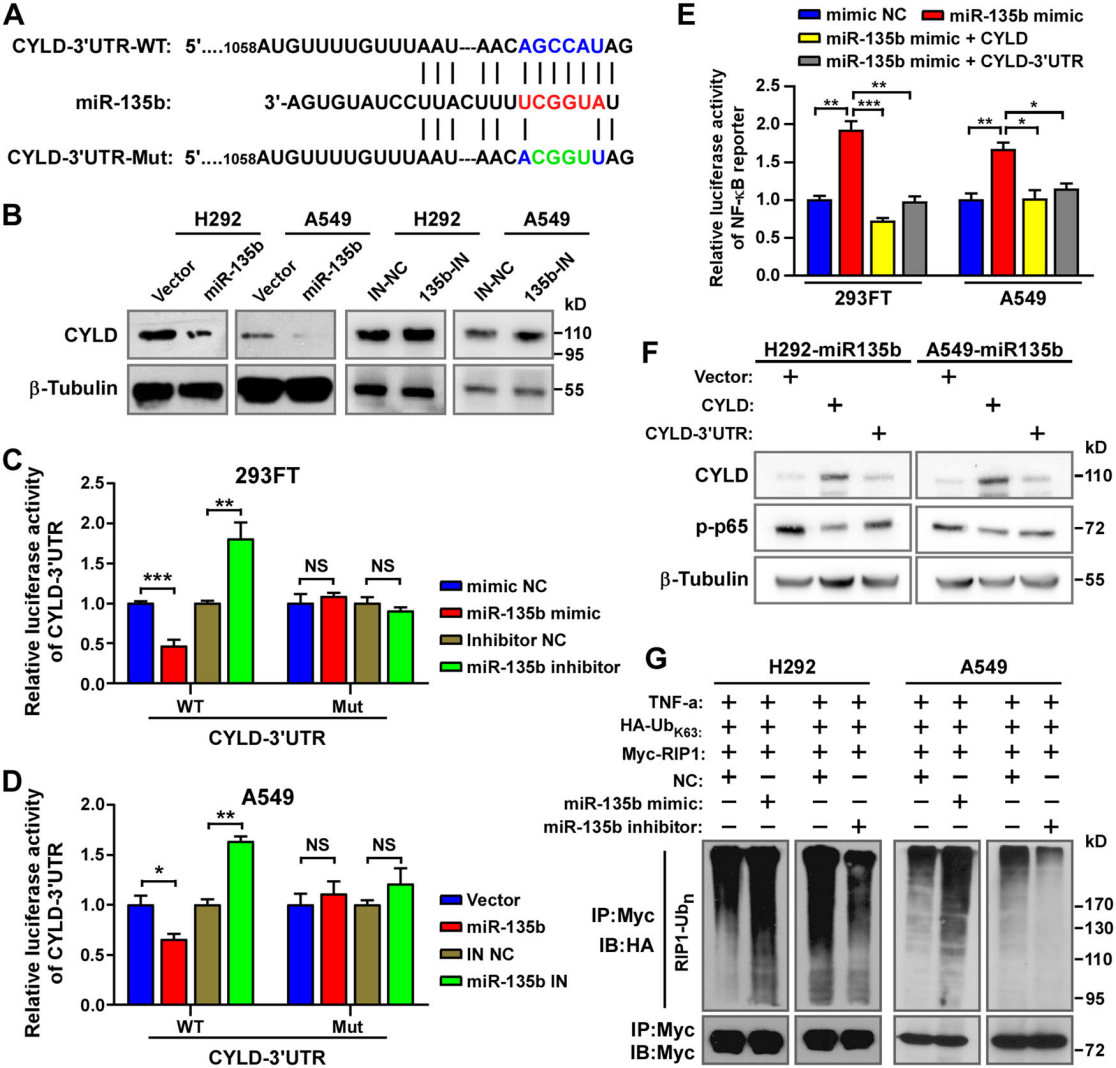
miR-135b directly targets the deubiquitinase CYLD to activate NF-κB signaling. **A** Predicted miR-135b target sequence in CYLD-3′ UTR (WT) and mutant containing 4 mutated nucleotides in the seed sequence of CYLD-3′ UTR (CYLD-3′UTR-Mut). **B** Western blotting analysis of CYLD expression in the indicated cells. β-Tubulin served as a loading control. **C**, **D** The activity of the CYLD-3′ UTR-WT and CYLD-3′ UTR-Mut luciferase reporter gene in indicated cells. **E** Activity of the NF-κB luciferase reporter gene in the indicated cells transfected with NC, miR-135 mimics and miR-135 mimics plus CYLD gene (without 3′ UTR) or CYLD-3′ UTR. **F** Western blotting analysis of p-p65 and CYLD in the indicated cells transfected with vector, CYLD, or CYLD-3′ UTR. β-Tubulin served as a loading control. **G** Western blotting analysis of K63-linked poly-Ub of RIP1 in H292 and A549 cells.

Next, how miR-135b activates NF-κB signaling was investigated. It is known that miRNAs inhibit translation and/or promote the degradation of their target RNAs. To activate NF-κB, miR-135b might inhibit one/multiple negative regulator(s) of NF-κB signaling. By employing algorithms from TargetScan Human 7.2 (http://www.targetscan.org/vert_72/), miRcode (http://www.mircode.org/index.php) and miRWalk (http://mirwalk.umm.uni-heidelberg.de/), the putative NF-κB signaling negative regulators CYLD^12,29^, SPATA2^30,31^, and TNIP1^32^ were predicted as the potential targets of miR-135b (Fig. 5A and data not shown). Western blotting assays showed that overexpression of miR-135b reduced only CYLD protein levels, and inhibition of miR-135b had the opposite effect in NSCLC cells (Fig. 5B and data not shown). Next, we constructed wild-type (WT) and mutated (Mut) CYLD-3′UTR luciferase reporters (Fig. 5A). The luciferase activity of the CYLD-3′UTR-WT reporter was significantly reduced by overexpression of miR-135b but increased by inhibition of miR-135b. In contrast, the activity of the CYLD-3′UTR-Mut reporter was barely affected (Fig. 5C, D). These data prove that CYLD is a direct target of miR-135b.

Furthermore, we explored whether targeting the CYLD-3′UTR and inhibiting the expression of CYLD could account for the effects of miR-135b on NF-κB signaling activation. As expected, rescuing the expression of CYLD by overexpression CYLD dramatically reversed the activation of NF-κB signaling by miR-135b, as indicated by the decreased NF-κB reporter luciferase activity (Fig. 5E) and reduced phosphorylation of p65 in miR-135b overexpressing NSCLC cells (Fig. 5F). Additionally, overexpression of the CYLD-3′UTR also reversed the effects of miR-135b on NF-κB signaling (Fig. 5E, F), as the CYLD-3′UTR construct can compete with endogenous CYLD mRNA for miR-135b binding, thus exhausting miR-135b to rescue CYLD expression (Fig. 5F). These results further proved that miR-135b directly targets the CYLD-3′UTR. CYLD is a deubiquitinase that removes K63-linked polyubiquitin chains from RIP1 to repress NF-κB signaling^12,29^, thus we examined the effect of miR-135b on the ubiquitination of RIP1. As shown in Fig. 5G, overexpressing miR-135b increased while inhibiting miR-135b decreased the K63-linked polyUb level of RIP1. Therefore, we concluded that miR-135b activates NF-κB signaling by directly targeting the deubiquitinase CYLD.

### Rescuing the expression of CYLD reversed the effects of miR-135b on cellular behaviors

**Fig. 6:**
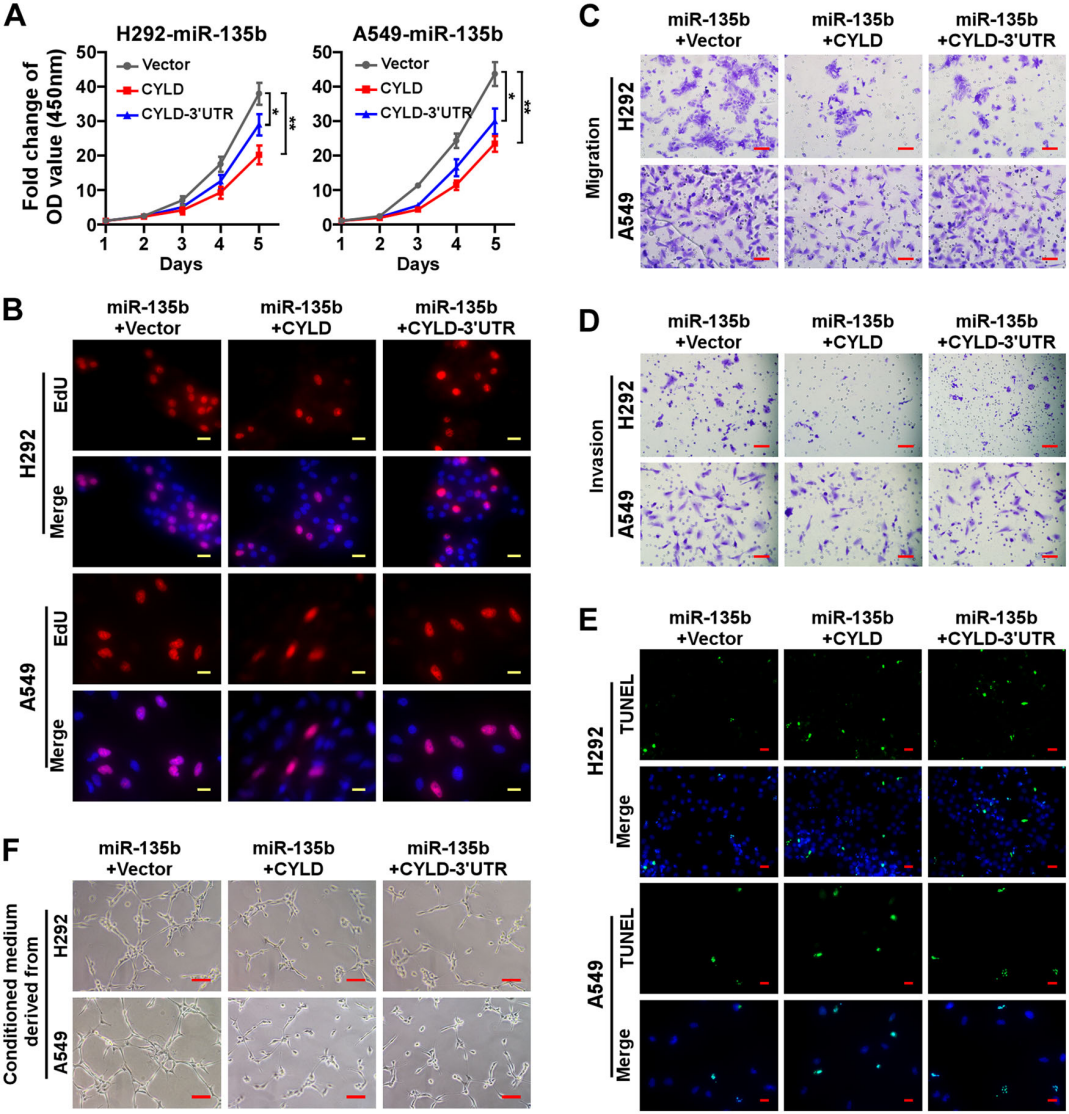
Rescuing the expression of CYLD reversed the effects of miR-135b on cellular behaviors. **A** The CCK-8 assay analysis of cell growth in the indicated stable cell lines. **B** Representative pictures of EdU staining. EdU was labeled with TRITC (red) and cells were counterstained with DAPI. **C**, **D** Representative pictures of cell migration (**C**) and invasion (**D**) in the indicated cells analyzed by transwell assay. Scale bars, 100 μm. **E** Representative pictures of cell apoptosis after treatment with cisplatin for 24 h analyzed by TUNEL staining (green). Cells were counterstained with DAPI. Scale bars, 20 μm. **F** Representative images of tubule formation by HUVECs cultured in Matrigel-coated plates with conditioned medium collected from the indicated NSCLC cells. Scale bars, 50 μm.

The abovementioned results prove that CYLD is a bona fide target of miR-135b mechanistically; hence, upregulation of CYLD would impair the effects of miR-135b on cellular behaviors. To prove this notion, we rescued CYLD in miR-135b overexpressing H292 and A549 cells by introducing CYLD or CYLD-3′UTR constructs. Rescuing the expression of CYLD significantly inhibited cell growth and proliferation as indicated by CCK-8 and EdU incorporation assays (Figs. 6A, B, and S4A). Moreover, overexpressing CYLD or the CYLD-3′UTR reduced migration, invasion, and survival, as evidenced by transwell assays and TUNEL staining, respectively (Figs. 6C–E and S4B–D). Tube formation assays showed that rescue the expression of CYLD inhibited the capillary tube formation of HUVECs (Fig. 6F).

### MiR-135b promotes tumor progression in vivo

**Fig. 7:**
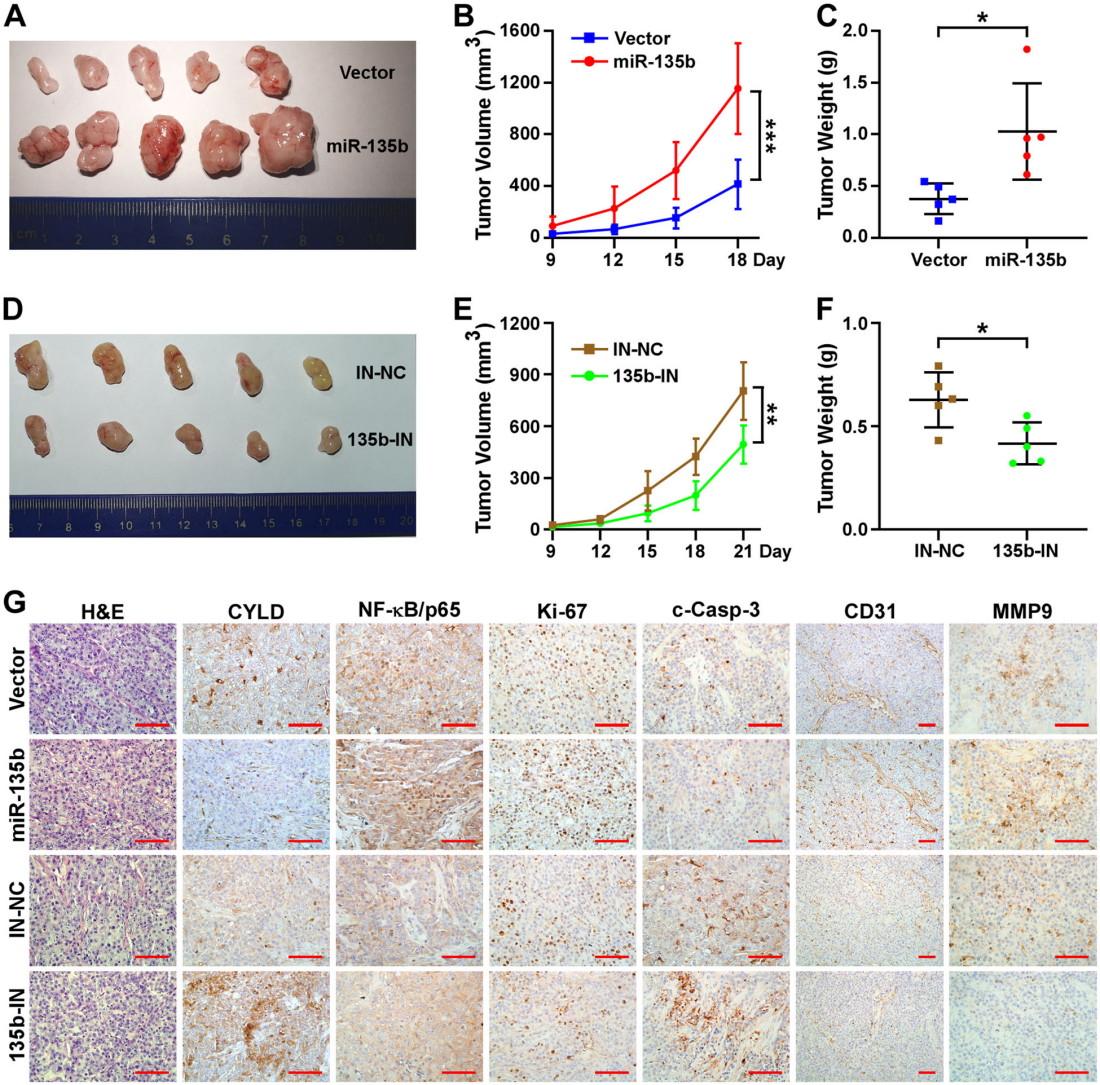
miR-135b promotes tumor progression in vivo. **A**, **D** Representative images of tumors from all of the mice in each group. **B**, **E** Growth curve of the tumor volumes measured on the indicated days. **C**, **F** Quantification of tumor weights. Error bars represent the mean ± SD. *p < 0.05; **p < 0.01; ***p < 0.001, two-tailed, unpaired *t*-test. **G** Representative pictures of H&E staining and IHC staining of CYLD, NF-kB/p65, Ki-67, cleaved-Casp-3, CD31 and MMP9 of the indicated group of tumors. Scale bars, 100 μm.

To further prove the tumor-promoting roles of miR-135b in NSCLC in vivo, a xenografted tumor model that was generated by subcutaneously implanting the indicated H292 cells into nude mice was employed. Consistent with the in vitro assay results, the tumor growth and tumor weight of the group with miR-135b overexpression were dramatically increased compared to the control group (Fig. 7A–C). Consistently, inhibiting miR-135b significantly impaired tumor growth and reduced tumor weight (Fig. 7D–F). Moreover, H&E and IHC staining proved that overexpression of miR-135b inhibited CYLD expression, activated NF-κB signaling (more nuclear staining of p65), and promoted cell proliferation (more Ki-67-positive cells), anti-apoptosis (less c-Casp-3), angiogenesis (more CD31-positive cells and microvascular structures) and invasiveness (increased MMP9) in vivo. Inhibiting miR-135b produced the opposite results (Fig. 7G).

### IL-6/STAT3 transactivates miR-135b in NSCLC

**Fig. 8:**
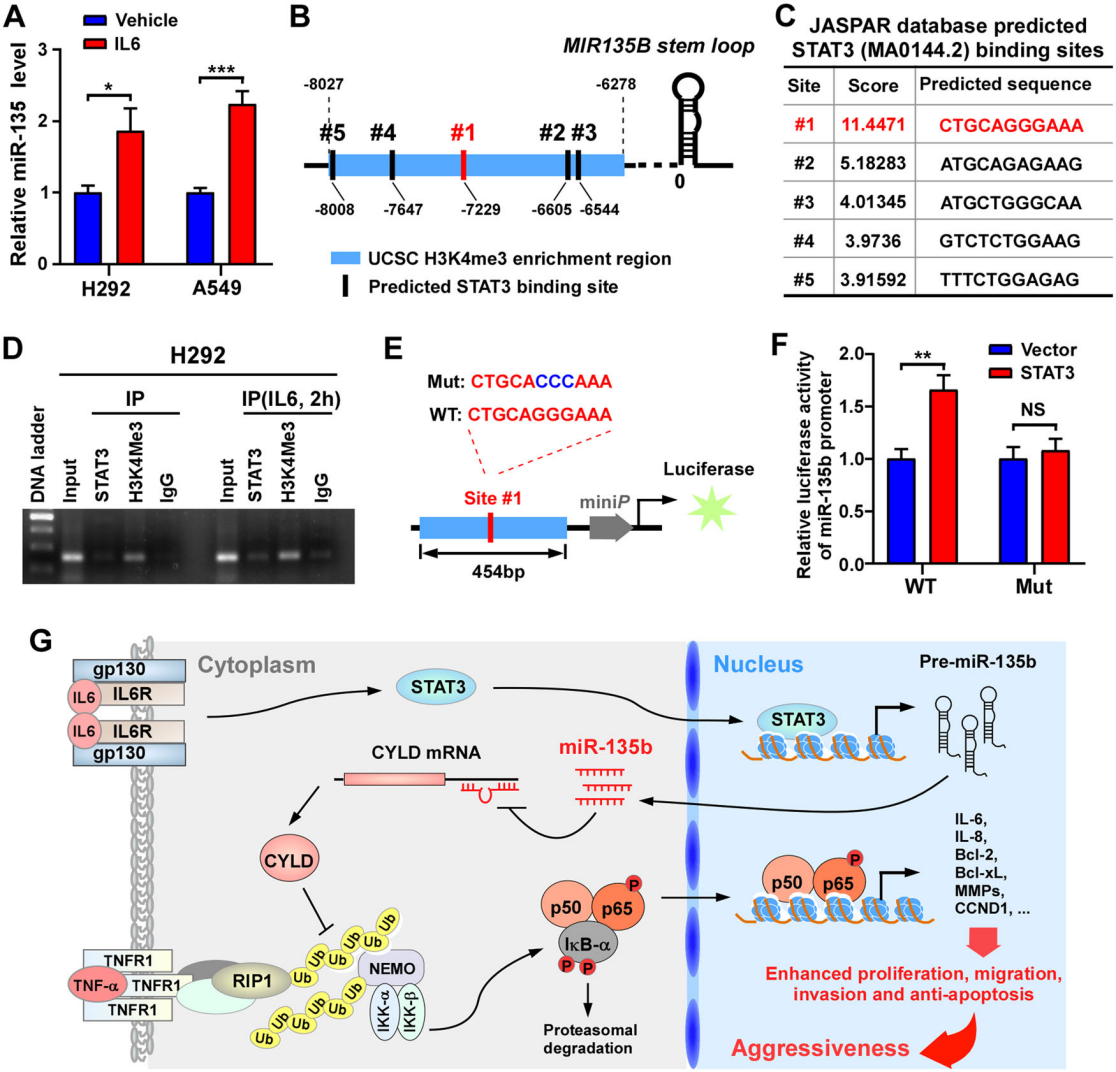
IL-6/STAT3 transactivates the expression of miR-135b in NSCLC. **A** Real-time PCR measuring the expression of miR-135b in H292 and A549 cells treated with human recombinant IL-6 (10 ng/ml, 6 h). **B**, **C** Schematic illustration of the arrangement of the promoter region of miR-135b and the top 5 conserved STAT3-binding sites predicted by the JASPAR database. **D** ChIP-PCR assay analyzing the enrichment of STAT3 and H3K4Me3 in the promoter of miR-135b. **E** Schematic illustration of the construction of miR-135b promoter luciferase reporters containing a region around site #1 with the STAT3 binding site intact (WT) or mutated (Mut). **F** The effects of STAT3 on the activity of the WT or Mut miR-135b promoter-luciferase reporter in 293FT cells. **G** Schematic representation of the STAT3/miR-135b/NF-κB signaling that promoting aggressiveness in NSCLC.

Although miR-135b has been reported to be upregulated in multiple cancers, the underlying mechanism has seldom been explored. Previously, Hironori et al. reported that IL-6 provokes the expression of miR-135b and its host gene LEMD1 in lymphoma^24^; however, whether STAT3 binds the miR-135b promoter region to activate its expression has not been proven. Consistently, we found that IL-6 treatment increased the expression of miR-135b in NSCLC cells (Fig. 8A). H3K4me3 is a highly conserved histone modification and a hallmark of active genes distributed along with promoter and transcription start site (TSS) regions^33,34^. By retrieving the UCSC Genome Browser (http://genoime.ucsc.edu/), a genomic region located from approximately 6278 to 8027 bp ahead of *MIR135B* stem-loop was identified to have enrichment of the H3K4me3 histone modification (Fig. 8B). Further analysis via the JASPAR database (http://jaspar.genereg.net/) predicted five conserved STAT3-binding sites in this H3K4me3-enriched region. Site 1 (#1) was the most likely STAT3-binding site and had a much higher score than the other sites (Fig. 8B, C). Hence, we analyzed the enrichment of STAT3 at site #1 by chromatin immunoprecipitation (ChIP) and PCR assays. The results showed that both H3K4me3 and STAT3 were enriched in site #1 and that there was increased enrichment upon IL-6 treatment (Fig. 8D). Moreover, miR-135b promoter luciferase reporters containing a region around site #1 with the STAT3-binding site intact (WT) or mutated (Mut) were constructed (Fig. 8E). Overexpression of STAT3 significantly increased the luciferase activity of the WT reporter, but had little effect on the Mut reporter (Fig. 8E). On the basis of these results, we conclude that IL-6/STAT3 transcriptionally elevates miR-135b.

## Discussion

In the current research, we found that miR-135b functionally promoted cell proliferation, migration, invasion, anti-apoptosis, and angiogenesis in vitro and in vivo. Moreover, mechanistically we identified CYLD as a novel target of miR-135b. miR-135b targeted the 3′UTR of CYLD and inhibited CYLD expression, which further potentiated the activation of NF-κB signaling. Our results indicate that miR-135b plays a tumor-promoting role in NSCLC.

Many studies have shown miR-135b elevation in different types of tumors^25,35,36^, but the underlying mechanism by which miR-135b is upregulated has seldom been investigated. Here, we proved that the IL-6/STAT3 axis transcriptionally upregulates miR-135b. IL-6 treatment significantly increased miR-135b expression. Via ChIP and luciferase reporter assays, we identified a potential promoter and validated a bioactive STAT3-binding site that activates miR-135b upstream of the miR-135b locus. Our findings are supported by previous reports. Hironori et al. also found that IL-6 provokes the expression of miR-135b and its host gene LEMD1 in anaplastic large-cell lymphoma (ALCL), and they identified STAT3-binding sites both upstream and downstream of the miR-135b locus^24^. However, miR-135b was found to be transcribed in the reverse direction of LEMD1, and the identified STAT3-binding sites were on the reverse strand. Hence, these sites might not transactivate miR-135b but its host gene LEMD1. Moreover, a study from Uri Rozovski and colleagues also stated that STAT3 regulates miR-135b in chronic lymphocytic leukemia. In addition, they described that the promoter region of miR-135b was located at 205416952–205452990 on chromosome 1q^37^, which contains the STAT3-binding region we identified. According to these two studies and ours, the STAT3-binding site that activates miR-135b identified in the current study is reliable.

Crosstalk between NF-κB and STAT3 signaling exists in a variety of cancers. NF-κB and STAT3 can bind the same promoters/enhancers and share many downstream genes^17^. The NF-κB/IL-6/STAT3 axis is well established. NF-κB transcriptionally activates IL-6 and then IL-6 interacts with the receptor to facilitate the activation of STAT3^38,39^. STAT3 enhanced acetyltransferase p300-mediated RelA acetylation and interfered with NF-κB nuclear export, thereby prolonging NF-κB nuclear retention and maintaining constitutive NF-κB activity^40^. Although STAT3 can also activate NF-κB, the reported mechanisms are relatively few. Here, we identified that IL-6/STAT3 upregulates miR-135b, subsequently activating NF-κB signaling, which indicates that STAT3 acts upstream of NF-κB signaling. Hence, our findings provide a new positive feedback loop of the IL-6/STAT3/miR-135b/NF-κB signaling transduction axis in NSCLC.

In addition to IL-6, other inflammatory cytokines have also been reported to upregulate miR-135b. Previously, Ching-Wen Lin et al. showed that TNF-α stimulates the expression of miR-135b in NSCLC^25^. Using transgenic mice, organoids, and immortalized cell lines, Tae-Su Han et al. proved that miR-135b is induced by IL-1 in gastric cancer. Moreover, they defined miR-135b as an inflammation-induced miRNA^20^. Surprisingly, aspirin, a well-known anti-inflammatory medicine, reduce hypoxia-enhanced numbers of exosomes and levels of exosomal miR-135b, thereby weakening the proliferation, migration, and angiogenesis of A549 cells^41^. These findings, in combination with ours, emphasize a close relationship between miR-135b and inflammation, and miR-135b might be a pivotal mediator in inflammation-related cancer progression.

MiR-135b has been proven to target multiple tumor suppressors in different types of cancer, resulting in cancer progression and treatment resistance. In osteosarcoma, miR-135b promotes cell proliferation and invasion by targeting FOXO1^42^ and stimulates recurrence and lung metastasis by targeting TET3 and multiple negative regulators of the Wnt/β-Catenin, including APC, CK1α, GSK3β, and β-TrCP^43^. miR-135b directly targets the BMAL1 3′-UTR and thereby promotes tumorigenesis and resistance to gemcitabine in pancreatic cancer^44^. By targeting the FIH1 3′-UTR and thereby potentiating HIF-1, exosomal miR-135b shed from hypoxic multiple myeloma cells enhances angiogenesis^45^. Moreover, Ching-Wen Lin et al. reported that miR-135b targets LZTS1 and negative regulators of the Hippo pathway, including LATS2, NDR2, MOB1, and β-TrCP, leading to lung cancer metastasis^25^. These findings, in addition to those from our current study, suggest that agents targeting miR-135b should exhibit favorable pharmacokinetics for cancer therapy. However, naked miRNAs and antisense oligonucleotides (ASOs) are easily degraded by ribonucleases and lack tumor-targeting ability^46,47^. Recently nanoparticles have arisen as an attractive option for tumor-targeted delivery of miRNAs and antagomiRs^47^. It is worthwhile to design nanomedicines targeting miR-135b for further preclinical and clinical studies of cancer treatment.

Overall, in this study, we found that miR-135b is frequently elevated and its elevation is associated with poor prognosis in NSCLC. IL-6/STAT3 axis activates and increases the expression of miR-135b. Upregulated miR-135b targets the 3′UTR of CYLD, inhibits the expression of CYLD, and subsequently activates NF-κB and its downstream factors, which ultimately results in enhanced cell proliferation, migration, invasion, anti-apoptosis, and angiogenesis and confers aggressiveness of NSCLC (Fig. 8G). Overall, miR-135b could be a therapeutic target for NSCLC.

## Materials and methods

### Patients’ information and tissue specimens

A cohort of 128 paraffin-embedded, archived NSCLC specimens obtained from Tianjin Medical University Cancer Institute and Hospital. The patients were diagnosed between December 2012 and January 2014 followed up until April 2018. Patient consent was informed and written. Patient consent and approval from the Institutional Research Ethics Committee of Tianjin Cancer Institute and Hospital were obtained for the use of the clinical materials for research purposes. The clinicopathological characteristics of patients are summarized in Table S1.

### Cell culture

The human lung cancer cell lines H1299, H1975, H292, H358, H460, H520, A549, PC-9, and Calu-3 were cultured in complete RPMI-1640 medium (Gibco, Grand Island, USA). Human umbilical vein endothelial cells (HUVECs) and 293FT cells were cultured in a complete DMEM medium (Gibco). The complete RPMI-1640 and DMEM medium were supplemented with 10% FBS (BI), 1% non-essential amino acids, 100 U/mL penicillin, and 100 μg/mL streptomycin (Gibco). Human lung bronchus epithelial cell line BEAS-2B was cultured in BEGM medium (Lonza). All cells were maintained in a humidified incubator at 37 °C with 5% CO_2_.

### Plasmid constructs and miRNAs

The human *MIR135B* gene was PCR amplified from genomic DNA and cloned into a pSin-EF2-IRES-puro lentiviral vector. miR-135b inhibition (135b-IN) vector was constructed according to a previous report^24^ by inserting the TuD RNA into a pSuper-puro retroviral vector. pNF-κB-luci-reporter and pMSCV-CYLD^28^ were kindly gifted by Prof. Chuyong Lin, Sun-Yat Sen University Cancer Center. Luciferase reporter constructs, pmirGLO-CYLD-WT, and pmirGLO-CYLD-Mut, were acquired by ligating double-stranded oligonucleotides embodying the wild type or mutated miR-135b target sites from CYLD, respectively, into the SalI/NdeI sites of pmirGLO vector. For overexpressing STAT3, the STAT3 coding region was inserted into pCDNA3.1 vector subcloned from a plasmid^48^ gifted from Dr. Chao Jing, Tianjin Medical Cancer Institute & Hospital. A 454 bp fragment of the miR-135b promoter region (Fig. 8E) was cloned into a pTAL-Luc vector for constructing miR-135b promoter-luciferase reporter. miR-135b mimic/inhibitor and Negative Control (NC) oligo were synthesized by Ribobio Inc. (Guangzhou, Guangdong, China).

### Western blotting

Western blotting was conducted as previously reported^49^. Primary antibodies against Bcl-xl (1:1000, #2764, Cell Signaling Technology, USA), Bcl-2 (1:500, #2764, Cell Signaling Technology, USA), CCND1 (1:1000, TA801655, Origene, USA), CYLD (1:1000, #2978, Cell Signaling Technology, USA), β-tubulin (1:5000, RM2003, Ruikang, Beijing), NF-κB p65 (1:1000, #8242, Cell Signaling Technology, USA), Phospho-p65 (Ser536) (1:1000, #3033, Cell Signaling Technology, USA), IKKβ (1:1000, #2684, Cell Signaling Technology, USA), Phospho-IKKα/β (Ser176/180) (1:500, #2694, Cell Signaling Technology, USA), PARP (1:1000, #YM3132, Immunoway, USA), Cleaved Caspase-3(Asp175) (1:1000, #9661, Cell Signaling Technology, USA) were used.

### Bioinformatics analysis

RNA-seq data of LUAD and LUSC from the lung Pan-Cancer study of TCGA database (https://cancergenome.nih.gov/) were used to analyze the expression of miR-135b. The data sets were downloaded in 2014. EdgeR package was used to analyze the difference of miRNAs’ expression between NSCLC tissues and control normal lung tissues. Log_2_ FC ≥ 1, FDR *p*-value < 0.05 was used as the cut-off value for the volcano plots analysis. RNA array dataset GSE102287 was downloaded from the NCBI/GEO database (https://www.ncbi.nlm.nih.gov/gds/) and the expression of miR-135b between paired NSCLC tissues and control normal lung tissues was analyzed.

### RNA extraction and quantitative real-time PCR

RNA extraction and real-time PCR were performed as previously described^50^. U6 small nuclear RNA and GAPDH were used as an internal control for miR-135b and its correlative mRNA, respectively. The relative RNA levels were calculated according to the comparative Ct (ΔΔC_t_) method, where Ct represents the threshold cycle for each transcript. The specific primers used are listed in Table S2.

### CCK-8 assay

Cell viability was determined with the Cell Counting Kit-8 Solution Reagent (CCK8, Biyuntian, Beijing, China) according to the manufacturer’s instruction. Cells (500 cells/well) were plated into 96-well plates. CCK8 was added to a final concentration of 10% and incubated for 2 h. The optical density (OD) value was detected at a wavelength of 450 nm by a microplate reader (Thermo Scientific, USA). All experiments were performed in five biological duplicates at least three times.

### Colony formation assay

Cells were evenly counted and seeded into six-well plates (500 cells/well). The cell culture medium was refreshed every 2 days. After the culturing for 7–10 days, cells were fixed by ice-cold methanol for 15 min and dyed with 0.1% crystal violet for 15 min. A colony containing more than 50 cells was taken into account. Three replicate wells were conducted for each group at least three times.

### EdU incorporation assay

5 × 10^4^ cells were seeded in a 24-well plate. The assay was conducted using the Cell Light EdU DNA imaging Kit (RiboBio, China) according to the manufacturer’s instruction. Briefly, 50 μM EdU was added and the cells were cultured for an additional 2 h. Then the cells were fixed and stained. Immunofluorescence images were captured with a laser scanning microscope (Axio Imager.Z2, Carl Zeiss Co. Ltd., Germany). At least five fields were randomly selected to evaluate the proportion of the EdU-positive cells.

### TUNEL assay

Indicated stable cell lines from H292 and A549 were treated by cisplatin (DDP), 5 and 7.5 μg/ml, respectively, for 24 h, to induce cell apoptosis. Apoptotic cells were detected by TUNEL assay using the DeadEnd^TM^ Fluorometric TUNEL System (Promega, USA) following the instruction. The immunofluorescence images were captured using ZEN viewer software (Axio Imager.Z2, Carl Zeiss Co. Ltd.). Five randomly selected fields were viewed to evaluate the proportion of TUNEL positive cells.

### In vitro cell invasion and migration assay

The cell invasion and migration capability were assessed using 24-well transwell Boyden chambers covered by polycarbonate membranes with 8.0-μm pore sizes (BD Biosciences, USA). Cells suspended in 200 μl FBS-free medium were added to the upper chamber which were pre-coated with (for invasion assay) or without (for migration assay) matrigel, while 600 μl medium with 20% FBS was added to the lower chamber as chemotaxis. 24 h later, cells on the top of the chamber were removed and those on the bottom were fixed with a mixture solution of absolute methanol and acetic acid (methanol: acetic acid = 3:1) and stained with 0.1% crystal violet. 5 × 10^4^ and 1 × 10^5^ cells were seeded to each chamber for migration and invasion analysis respectively. The stained cells were quantified under a microscope (5 random fields per well, 200 × magnification).The mean numbers of cells per field were presented.

### Tube formation assay

Matrigel (BD Biosciences, USA) was thawed on the ice at 4 °C overnight. 200 μl of ice-cold matrigel was transferred into each well of a 24-well plate and polymerized for 2–3 h at 37 °C. The culture medium derived from NSCLC cell lines is namely conditioned medium (CM). 2 × 10^4^ HUVECs in 200 μl of CM were seeded to each well that was pre-coated with matrigel. The capillary tube structure was photographed under a ×100 bright-field microscope at 5 h of incubation.

### Immunofluorescence staining

4 × 10^4^ cells were seeded on coverslips (Thermo Fisher Scientific, USA) in 24-well plates overnight. Cells were treated with TNF-α (10 ng/ml) for 20 min and then stained as previously described^51^. Primary antibody against NF-κB p65 (1:100, sc-8008, Santa Cruz, USA) and TRITC-conjugated secondary antibody (1:500; Jackson Immuno-Research Laboratories, USA) were used. Cells were mounted with ProLong Diamond Anti-fade reagent with DAPI (Invitrogen, USA). Graylevel images were acquired under a laser scanning microscope (Axio Imager.Z2, Carl Zeiss Co. Ltd.).

### Luciferase reporter assay

8 × 10^4^ 293FT cells were seeded into each well of a 24-well plate and cultured overnight. 100 ng luciferase reporter plasmids or the control luciferase plasmid were co-transfected with 50 nM mimic NC, inhibitor NC, miR-135b mimic or miR-135b inhibitor (RiboBio, China). The Renilla luciferase plasmid (pRL-TK, Promega, USA) was co-transfected as a transfection control. The luciferase activity was measured 24 h after transfection using a Dual-Luciferase Reporter Kit (Promega, USA).

### Xenograft tumor model

Female BALB/C-nude mice (5-week-old) were purchased from Nanjing Biomedical Research Institute of Nanjing University (NBRI) and maintained in specific pathogen-free (SPF) conditions. All experimental procedures were in compliance with the guidelines of the Laboratory Animal Ethics Committee of Tianjin Medical University Cancer Institute and Hospital. The animals were grouped randomly. 6 × 10^6^ cells were subcutaneously injected into the areas of inguinal regions on every subject (5 mice per group). Tumor volume (*V*) was blinded monitored by measuring the length (*L*) and width (*W*) of the tumor with calipers and was calculated with the formula *V* = (*L* × *W*)×0.5. After mice were sacrificed, tumors were then removed, weighed, and fixed in 4% formalin for further study.

### In situ hybridization

Mature miR-135b was detected by miRNA in situ hybridization with the miRCURY LNA miRNA ISH Optimization Kits (FFPE) (QIAGEN, Germany). The assay was conducted with probes for human miR-135b, scramble, and U6 with a modified version of the manufacturer’s protocol for formalin-fixed paraffin-embedded tissue. The sections were examined and scored independently by two observers for two respects proportion of positively stained tumor cells and signal intensity. The proportion of tumor cells (P) was graded as follows: 0, 0–5% positive tumor cells; 1, 6–25% positive tumor cells; 2, 26–50% positive tumor cells; 3, 51–75% positive tumor cells; 4, 76–100% positive tumor cells. The staining intensity (*I*) was evaluated as follows: 0, no staining; 1, weak staining; 2, moderate staining; 3, strong staining. The staining index (SI) was calculated with the formula: SI = *P* × *I*. The SI mark that <4 (including 4) was deemed miR-135b-Low expression, while that more than 6 (including 6) was deemed miR-135b-High expression.

### Immunohistochemistry (IHC)

IHC was performed as described previously^52^. Antibodies against NF-κB p65 (1:200, #8242, Cell Signaling Technology, USA), CYLD (1:100, #2978, Cell Signaling Technology, USA), Ki67 (1:100, #9027,Cell Signaling Technology, USA), Cleaved Caspase-3 (Asp175) (1:50, #9661, Cell Signaling Technology, USA), CD31 (1:100, ab76533, Abcam, USA), MMP9 (1:100, #, Cell Signaling Technology, USA) were used.

### ChIP assay

ChIP assays were performed according to the manufacturer’s instructions using ChIP Assay Kit (#17-295, Millipore, USA). Briefly, cells were treated with IL-6 (50 ng/ml) or vehicle for 2 h and then with 1% formaldehyde to cross-link proteins to DNA. The cell lysates were sonicated to shear DNA to sizes of 200–500 bp. Equal aliquots of chromatin supernatants were separated and incubated with 1 μg anti-STAT3 (#9139, Cell Signaling Technology, USA), anti-H3K4me3 antibodies (#05-745 R, Sigma-Aldrich, USA) or an anti-IgG antibody (NC, #53484, Cell Signaling Technology, USA) overnight at 4 °C with rotation. After reverse cross-link of protein/DNA complexes to free DNA, PCR was performed using specific primers. The primer sequences were: Fwd, 5′-TCCTCCTTCTTCCTCTTCATTTTG-3′; Rev, 5′-CTTTCTCCTGCCCCCTCTC-3′.

### Statistical analysis

Statistical analysis was conducted using SPSS 20.0 statistical software package and GraphPad Prism 8.0 software. Mean ± SD was used to present the data from experiments. The differences between the two groups were assessed by the 2-tailed Student *t-*test. The *χ*^2^ test was used to access the relationship between miR-135b expression and clinicopathological characteristics. Survival curves were analyzed with the Kaplan–Meier method. Cox proportional hazards regression analysis was used to calculated hazards (HRs) and the 95% confidence intervals (CIs). A value of *P* < 0.05 was considered statistically significant.

## Supplementary information

Supplementary materials

Figure S1

Figure S2

Figure S3

Figure S4
