# Supplementary material for: STAT3/miR-135b/NF-κB axis confers aggressiveness and unfavorable prognosis in non-small-cell lung cancer
Source: Cell Death Dis. 2021 May 14;12(5):493. doi: 10.1038/s41419-021-03773-x (PMC8121828; doi:10.1038/s41419-021-03773-x)
Supplement: Supplementary file 1 — Supplementary materials [file 41419_2021_3773_MOESM1_ESM.pdf]

Supplementary Tables

Table S1. Clinicopathological characteristics of patients

Characteristics	No. （%）
**Gender**	
Male	48（37.5）
Female	80（62.5）
**Age(years)**	
<60	65（50.78）
>=60	63（49.22）
**Smoke**	
Yes	52（40.63）
NO	76（59.38）
**T classification**	
T1	75（58.59）
T2	49（38.28）
T3	2（1.56）
T4	2（1.56）
**N classification**	
N0	80（62.50）
N1	7（5.47）
N2	41（32.03）
**M classification**	
M0	127（99.22）
M1	1（0.78）
**Clinical stage**	
Ⅰ	75（58.59）
Ⅱ	11（8.59）
Ⅲ	41（32.03）
Ⅳ	1（0.78）
**Patient survival**	
Alive	99（77.34）
Deceased	29（22.66）
**Expression of miR-135b**	
Low expression	61（47.66）
High expression	67（52.34）

Table S2. DNA sequences of qRT-PCR primers

Primer name	Sequence
GAPDH_Fwd	GAAGGTGAAGGTCGGAGTCA
GAPDH_Rev	TTGAGGTCAATGAAGGGGTC
Bcl-2_Fwd	GACTGAGTACCTGAACCGGC
Bcl-2_Rev	CAGCCAGGAGAAATCAAACAG
Bcl-xL_Fwd	GTATTGGTGAGTCGGATCGC
Bcl-xL-Rev	TGCTGCATTGTTCCCATAGA
MMP9_Fwd	GGGACGCAGACATCGTCATC
MMP9_Rev	TCGTCATCGTCGAAATGGGC
A20_Fwd	GCGTTCAGGACACAGACTTG
A20_Rev	TTCATCATTCCAGTTCCGAGTATC
NFKBIA_Fwd	GTCAAGGAGCTGCAGGAGAT
NFKBIA_Rev	TCATGGATGATGGCCAAGT
VEGFC_Fwd	GTGTCCAGTGTAGATGAACTC
VEGFC_Rev	ATCTGTAGACGGACACACATG
IL-1β_Fwd	AGCTGATGGCCCTAAACAGA
IL-1β_Rev	CCTGAAGCCCTTGCTGTAGT
IL-6_Fwd	AGTGAGGAACAAGCCAGAGC
IL-6_Rev	CATTTGTGGTTGGGTCAGG
IL-8_Fwd	CGGAAGGAACCATCTCACTG
IL-8_Rev	AGCACTCCTTGGCAAAACTG

Table S3. Correlation between the clinicopathological features and expression of miR-135b

Characteristics	miR-135bexpression		χ^2 Test *P*-Value	Fisher Exact Test *P*-Value
	Low	High		
**Gender**				
Male	24	24	0.717	-
Female	37	43		
**Age(years)**				
<60	26	39	0.111	-
>=60	35	28		
**Smoke**				
Yes	28	24	0.041	
NO	33	43		
**T classification**				
T1	40	35	-	0.438
T2	19	30		
T3	1	1		
T4	1	1		
**N classification**				
N0	34	46	-	0.244
N1	5	2		
N2	22	19		
**M classification**				
M0	61	66	-	1.000
M1	0	1		
**Clinical stage**				
Ⅰ	32	43	-	0.165
Ⅱ	8	3		
Ⅲ	21	20		
Ⅳ	0	1		

Supplementary Figure Legends

Figure S1 Verification of miR-135b stably up- or downregulated NSCLC cell lines. (**A**) Real-time PCR measuring miR-135b level in miR-135 overexpressing cells. (**B**) Real-time PCR measuring miR-135b level in miR-135 inhibiting cells. Error bars represent the mean ± SD. **, *p*<0.01; ***, *p*<0.001, two-tailed, unpaired t-test.

Figure S2 miR-135b promotes cell migration, invasion, anti-apoptosis and angiogenesis in NSCLC *in vitro*. (**A, B**) Quantification of migrated (**A**) and invaded (**B**) cells in the indicated cell lines, related to Figure 3A-B. (**C**) Quantification of TUNEL positive cells in the indicated cell lines, related to Figure 3C. (**D**) Representative images of cells treated with cisplatin (DDP) taken under a bright field microscope. Scale bars, 50μm. (**E**) Western blotting analysis of the protein levels of PARP and cleaved-PARP in the indicated cells. β-Tubulin served as a loading control. (**F**) Quantification of migrated HUVECs, related to Figure 3D. Error bars represent the mean ± SD. *, *p*<0.05; **, *p*<0.01; ***, *p*<0.001, two-tailed, unpaired t-test.

Figure S3 miR-135b promotes nuclear translocation of NF-κB/p65. Representative images (A) and quantification (B) of p65 nuclear positive cells in indicated cells, related to Figure 4G. Scale bars, 20μm. Error bars represent the mean ± SD. *, *p*<0.05; **, *p*<0.01; ***, *p*<0.001, two-tailed, unpaired t-test.

Figure S4 Rescuing the expression of CYLD reversed the effects of miR-135b on cellular behaviors. (**A**) Quantification of EdU positive cells in indicated cell lines, related to Figure 6B. (**B, C**) Quantification of migrated (**B**) and invaded (**C**) cells in indicated cell lines, related to Figure 6C-D. (**D**) Quantification of TUNEL positive cells in indicated cell lines, related Figure 6E. Error bars represent the mean ± SD. *, *p*<0.05; **, *p*<0.01; ***, *p*<0.001, two-tailed, unpaired t-test.
