# Supplementary figures and images for: STAT3/miR-135b/NF-κB axis confers aggressiveness and unfavorable prognosis in non-small-cell lung cancer
Source: Cell Death Dis. 2021 May 14;12(5):493. doi: 10.1038/s41419-021-03773-x (PMC8121828; doi:10.1038/s41419-021-03773-x)

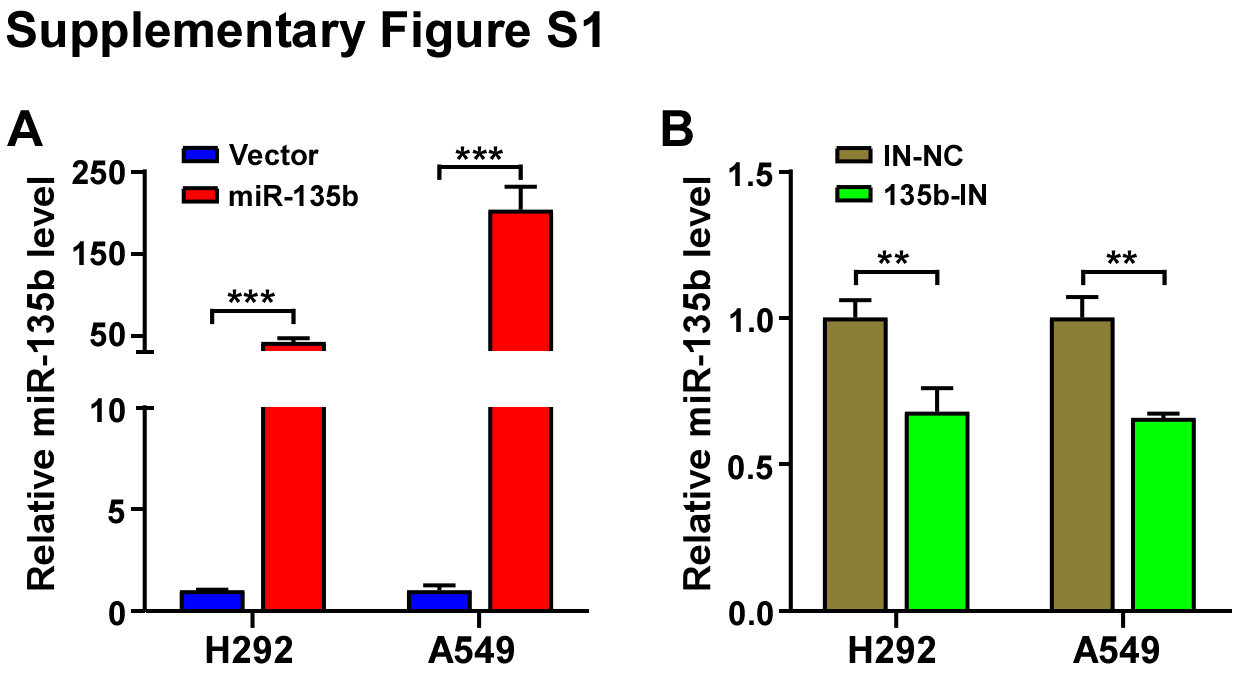

Supplement: Supplementary file 2 — Figure S1 [file 41419_2021_3773_MOESM2_ESM.tif]

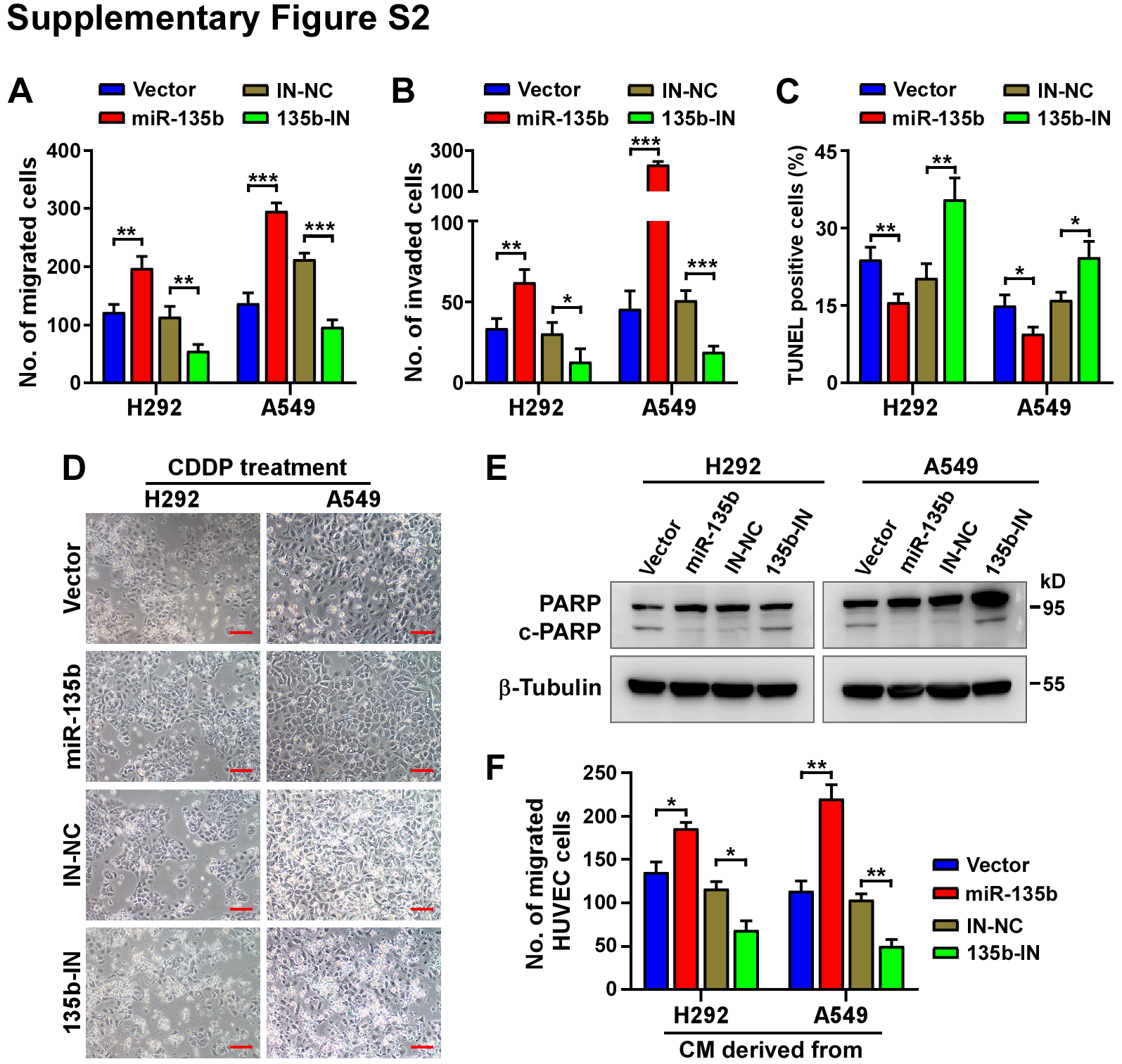

Supplement: Supplementary file 3 — Figure S2 [file 41419_2021_3773_MOESM3_ESM.tif]

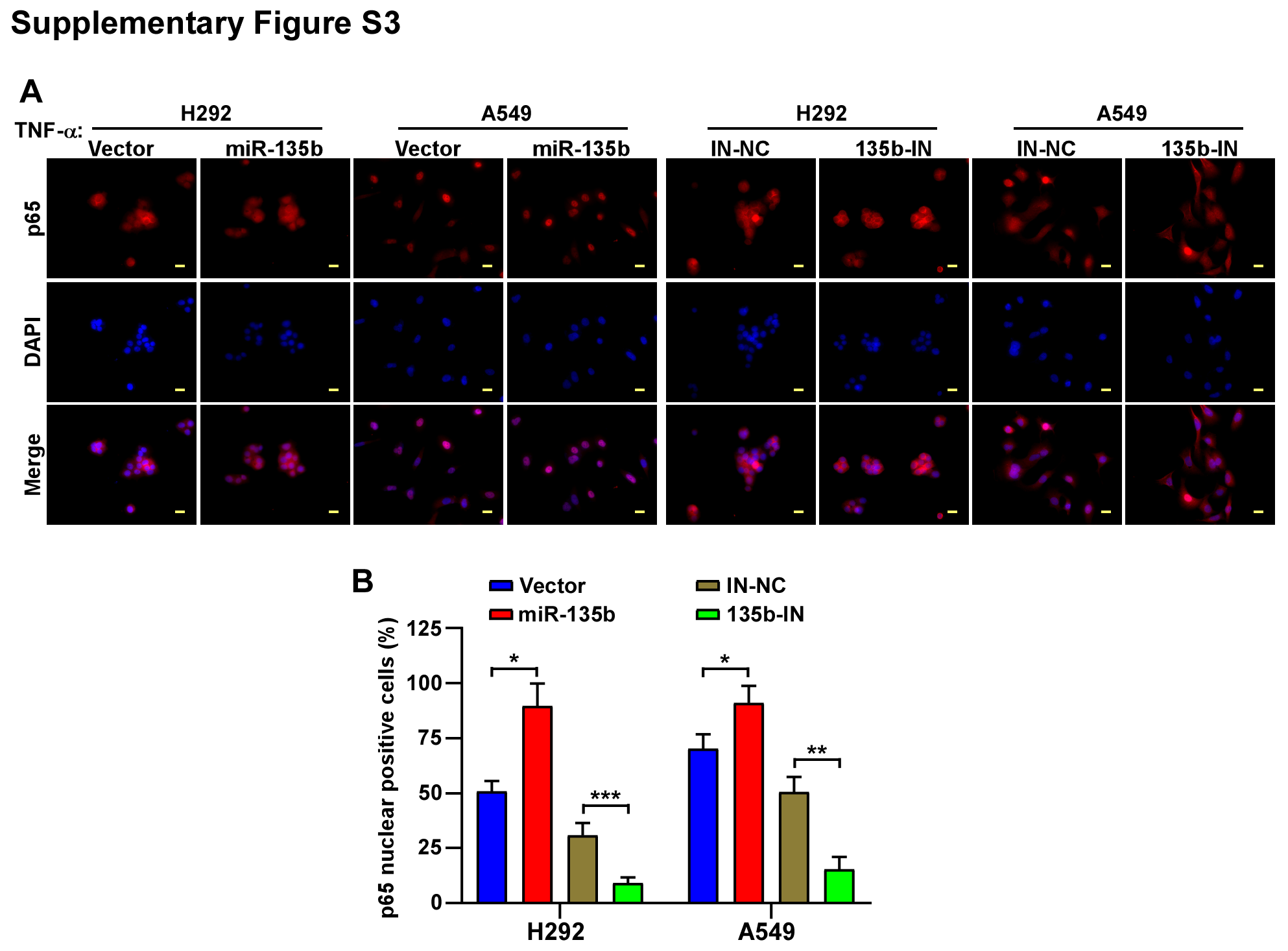

Supplement: Supplementary file 4 — Figure S3 [file 41419_2021_3773_MOESM4_ESM.tif]

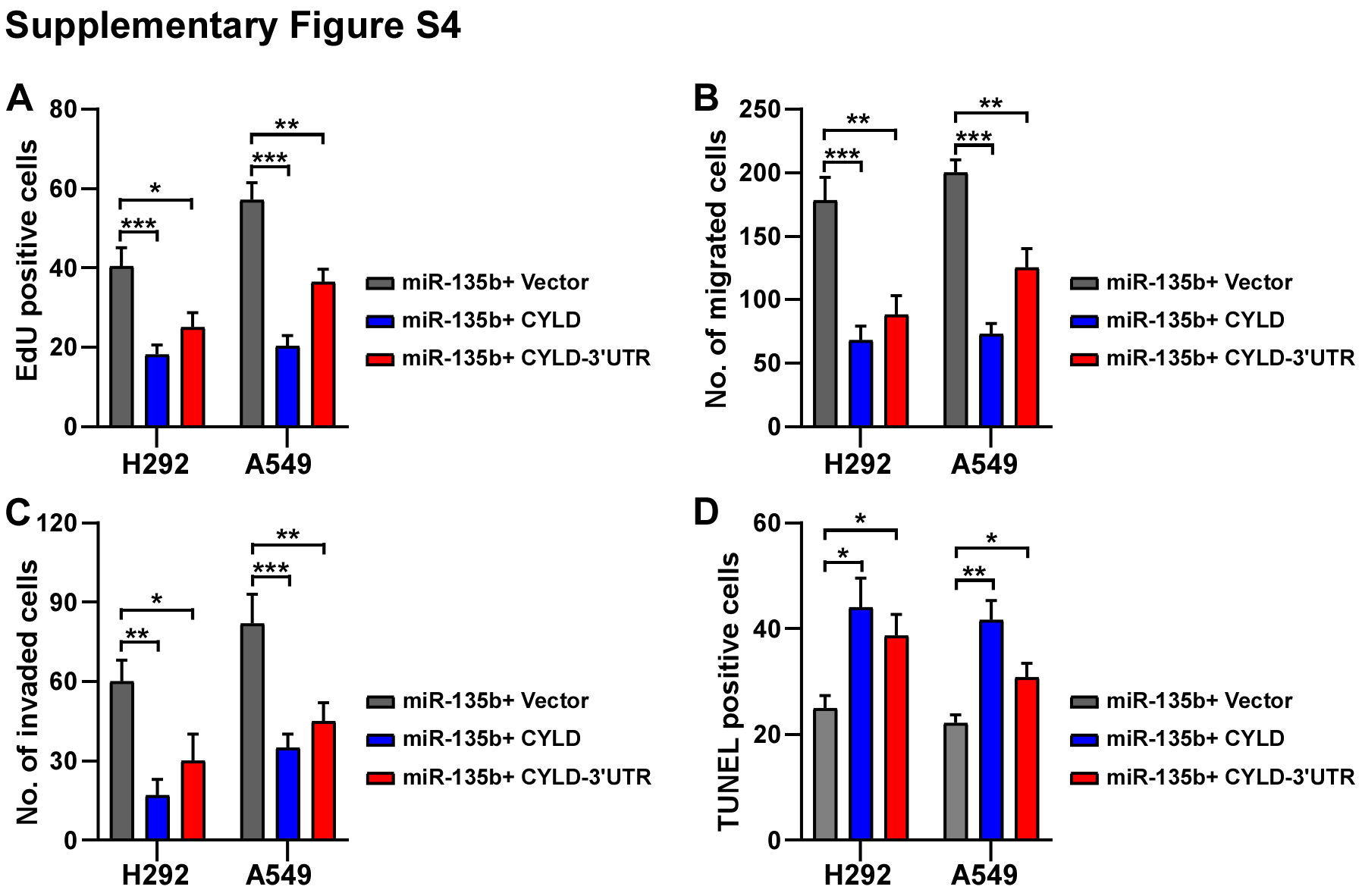

Supplement: Supplementary file 5 — Figure S4 [file 41419_2021_3773_MOESM5_ESM.tif]
